# Supplementary material for: Highly-automated, high-throughput replication of yeast-based logic circuit design assessments
Source: Synth Biol (Oxf). 2022 Oct 6;7(1):ysac018. doi: 10.1093/synbio/ysac018 (PMC9583850; doi:10.1093/synbio/ysac018)
Supplement: ysac018_Supp [file ysac018_supp.zip › suppl_data/SYNBIO-2021-042.tgz!/SYNBIO-2021-042/figures/gander_fig_three.pdf]

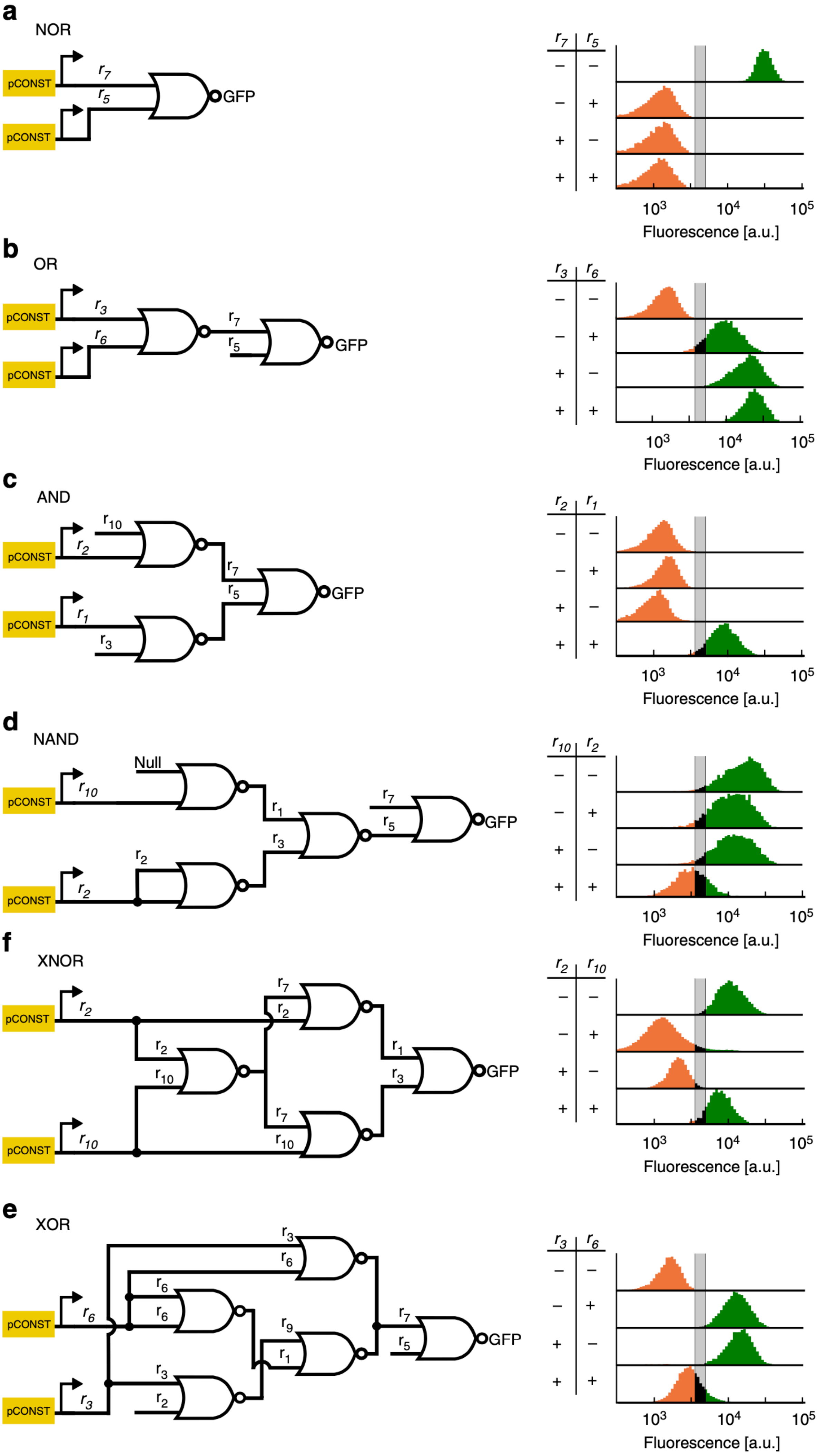

a
NOR
pCONST
pCONST
r7
r5
GFP
r7 r5
Fluorescence [a.u.]
b
OR
pCONST
pCONST
r3
r6
r7
r5
GFP
r3 r6
Fluorescence [a.u.]
c
AND
pCONST
pCONST
r10
r2
r1
r3
r7
r5
GFP
r2 r1
Fluorescence [a.u.]
d
NAND
Null
pCONST
pCONST
r10
r2
r1
r3
r7
r5
GFP
r10 r2
Fluorescence [a.u.]
f
XNOR
pCONST
pCONST
r2
r10
r7
r1
r3
GFP
r2 r10
Fluorescence [a.u.]
e
XOR
pCONST
pCONST
r6
r3
r9
r1
r2
r7
r5
GFP
r3 r6
Fluorescence [a.u.]
