# Supplementary material for: Highly-automated, high-throughput replication of yeast-based logic circuit design assessments
Source: Synth Biol (Oxf). 2022 Oct 6;7(1):ysac018. doi: 10.1093/synbio/ysac018 (PMC9583850; doi:10.1093/synbio/ysac018)
Supplement: ysac018_Supp [file ysac018_supp.zip › suppl_data/SYNBIO-2021-042.tgz!/SYNBIO-2021-042/figures/harmonized_protocol.pdf]

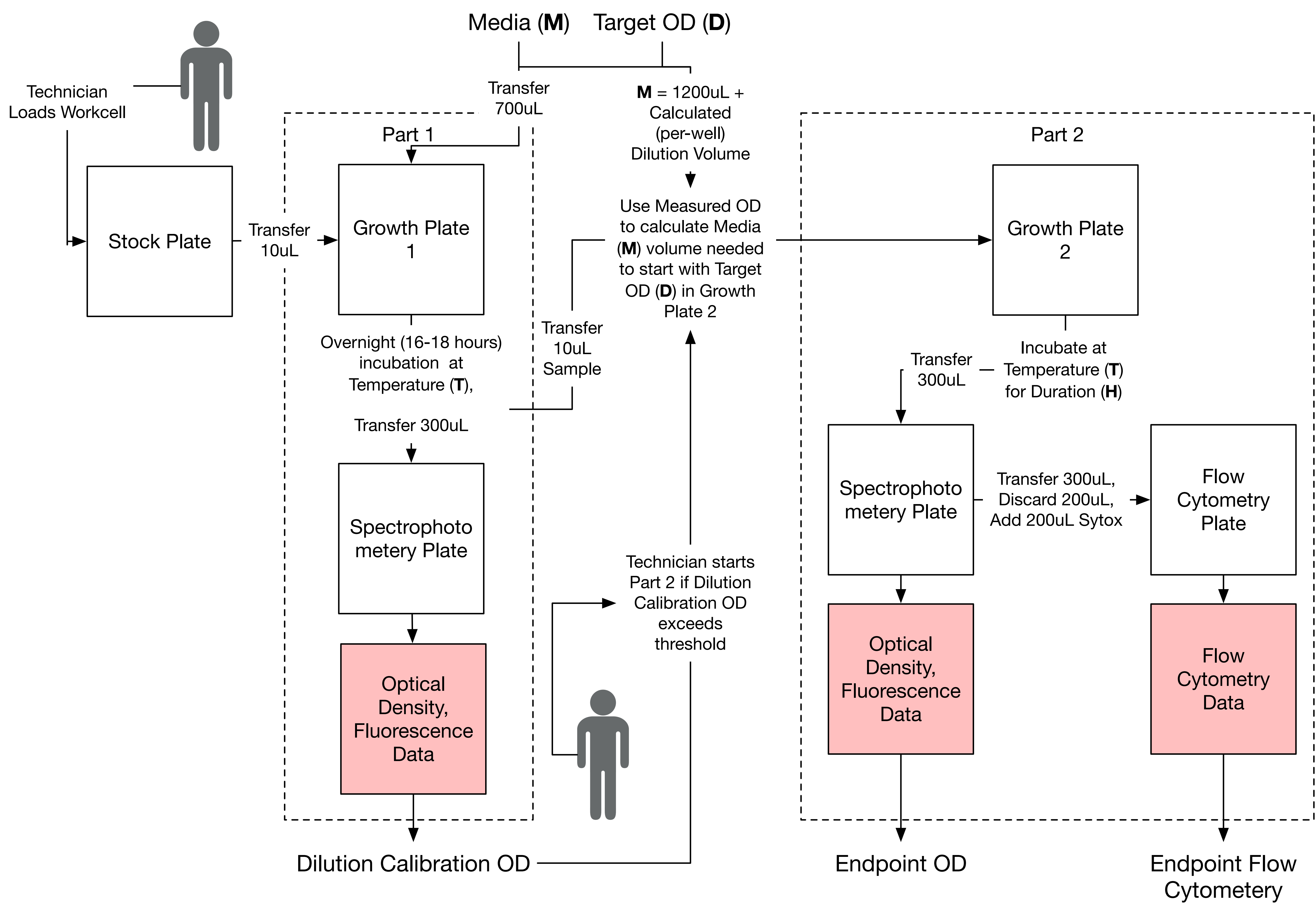

Media (**M**)
Target OD (**D**)
Technician Loads Workcell
Transfer 700uL
M = 1200uL + Calculated (per-well) Dilution Volume
Part 1
Part 2
Stock Plate
Transfer 10uL
Growth Plate 1
Use Measured OD to calculate Media (**M**) volume needed to start with Target OD (**D**) in Growth Plate 2
Growth Plate 2
Overnight (16-18 hours) incubation at Temperature (**T**),
Transfer 10uL Sample
Transfer 300uL
Incubate at Temperature (**T**) for Duration (**H**)
Transfer 300uL
Spectrophotometery Plate
Spectrophotometery Plate
Transfer 300uL, Discard 200uL, Add 200uL Sytox
Flow Cytometry Plate
Technician starts Part 2 if Dilution Calibration OD exceeds threshold
Optical Density, Fluorescence Data
Optical Density, Fluorescence Data
Flow Cytometry Data
Dilution Calibration OD
Endpoint OD
Endpoint Flow Cytometery
