# Supplementary material for: Highly-automated, high-throughput replication of yeast-based logic circuit design assessments
Source: Synth Biol (Oxf). 2022 Oct 6;7(1):ysac018. doi: 10.1093/synbio/ysac018 (PMC9583850; doi:10.1093/synbio/ysac018)
Supplement: ysac018_Supp [file ysac018_supp.zip › suppl_data/SYNBIO-2021-042.tgz!/SYNBIO-2021-042/figures/side_scatter_saturation_positive_controls.pdf]

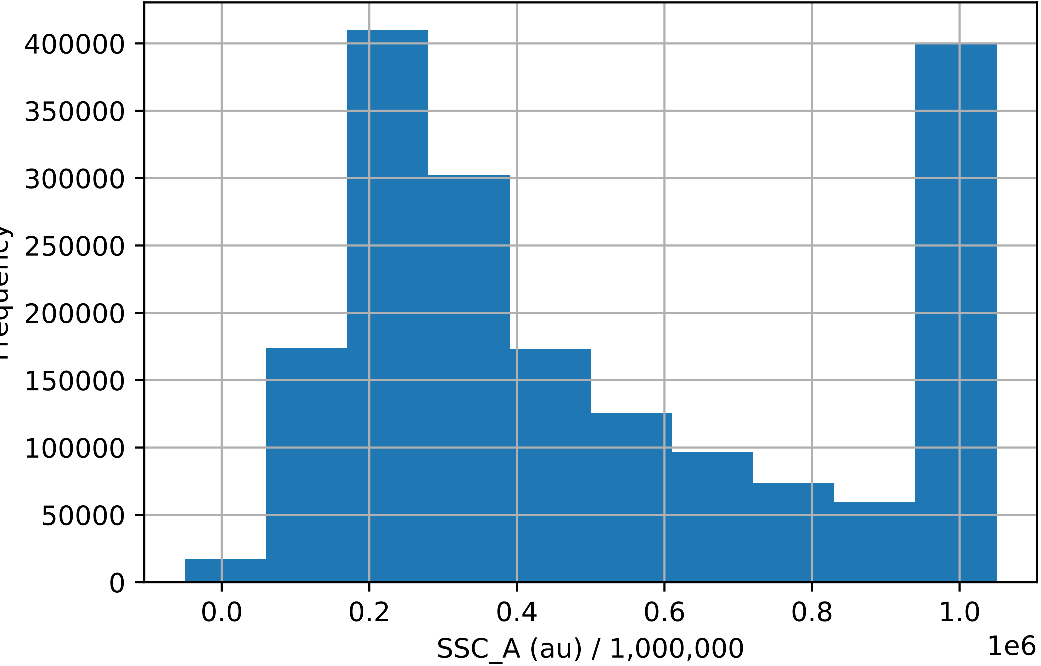

400000
350000
300000
250000
200000
150000
100000
50000
0
0.0
0.2
0.4
0.6
0.8
1.0
1e6
SSC_A (au) / 1,000,000
