# Supplementary material for: Highly-automated, high-throughput replication of yeast-based logic circuit design assessments
Source: Synth Biol (Oxf). 2022 Oct 6;7(1):ysac018. doi: 10.1093/synbio/ysac018 (PMC9583850; doi:10.1093/synbio/ysac018)
Supplement: ysac018_Supp [file ysac018_supp.zip › suppl_data/SYNBIO-2021-042.tgz!/SYNBIO-2021-042/figures/saturation_heatmap.pdf]

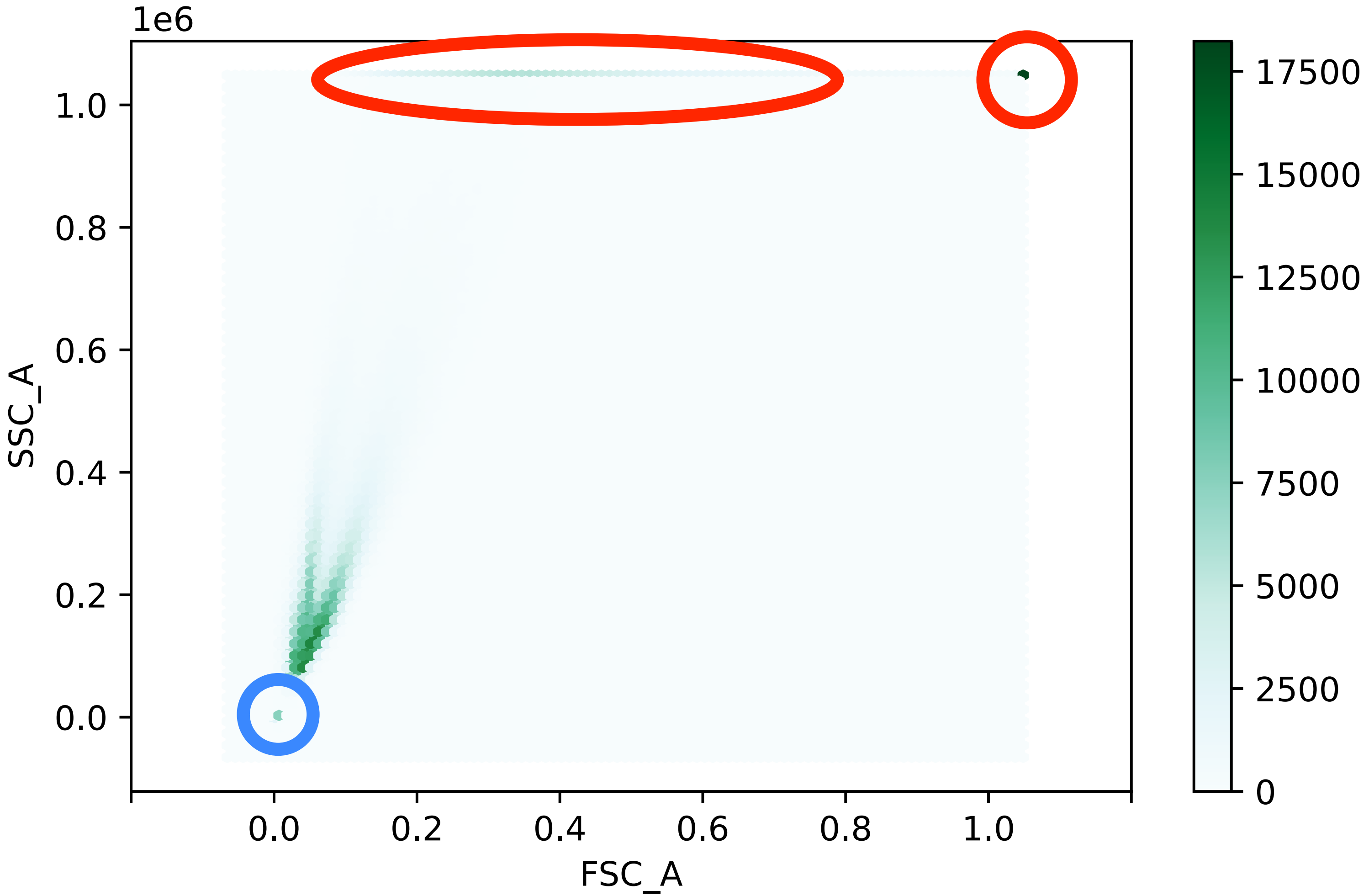

1e6
1.0
0.8
0.6
0.4
0.2
0.0
SSC_A
0.0
0.2
0.4
0.6
0.8
1.0
FSC_A
17500
15000
12500
10000
7500
5000
2500
0
