# Supplementary material for: Highly-automated, high-throughput replication of yeast-based logic circuit design assessments
Source: Synth Biol (Oxf). 2022 Oct 6;7(1):ysac018. doi: 10.1093/synbio/ysac018 (PMC9583850; doi:10.1093/synbio/ysac018)
Supplement: ysac018_Supp [file ysac018_supp.zip › suppl_data/SYNBIO-2021-042.tgz!/SYNBIO-2021-042/figures/correctness_table.pdf]

		All Growth Conditions		
gate	input	p(correct)	n(correct)	n
AND	00	0.99	178	180
AND	01	0.98	172	175
AND	10	0.99	166	167
AND	11	0.96	135	141
NAND	00	0.96	227	237
NAND	01	0.97	261	269
NAND	10	0.95	233	245
NAND	11	0.42	99	235
NOR	00	1.00	265	266
NOR	01	0.98	234	238
NOR	10	0.99	252	254
NOR	11	0.42	110	262
OR	00	0.99	189	191
OR	01	0.49	87	177
OR	10	0.02	4	188
OR	11	0.99	182	184
XNOR	00	0.95	200	211
XNOR	01	0.99	214	217
XNOR	10	0.99	213	215
XNOR	11	0.67	143	214
XOR	00	0.99	253	256
XOR	01	0.36	87	242
XOR	10	0.94	228	242
XOR	11	0.96	250	260
		18		5266
