# Supplementary material for: Highly-automated, high-throughput replication of yeast-based logic circuit design assessments
Source: Synth Biol (Oxf). 2022 Oct 6;7(1):ysac018. doi: 10.1093/synbio/ysac018 (PMC9583850; doi:10.1093/synbio/ysac018)
Supplement: ysac018_Supp [file ysac018_supp.zip › suppl_data/SYNBIO-2021-042.tgz!/SYNBIO-2021-042/figures/sequencing_analysis.pdf]

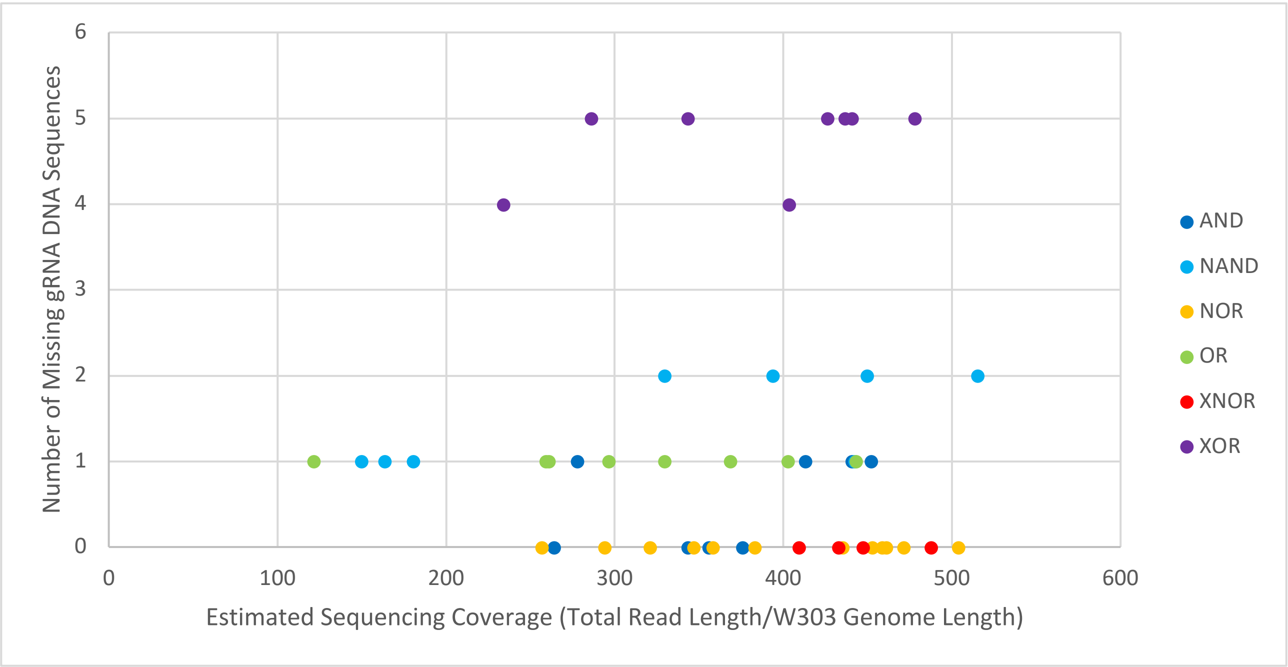

Number of Missing gRNA DNA Sequences
6
5
4
3
2
1
0
0
100
200
300
400
500
600
Estimated Sequencing Coverage (Total Read Length/W303 Genome Length)
AND
NAND
NOR
OR
XNOR
XOR
