# Supplementary material for: Highly-automated, high-throughput replication of yeast-based logic circuit design assessments
Source: Synth Biol (Oxf). 2022 Oct 6;7(1):ysac018. doi: 10.1093/synbio/ysac018 (PMC9583850; doi:10.1093/synbio/ysac018)
Supplement: ysac018_Supp [file ysac018_supp.zip › suppl_data/SYNBIO-2021-042.tgz!/SYNBIO-2021-042/figures/positive_control_clusters_fluorescence.pdf]

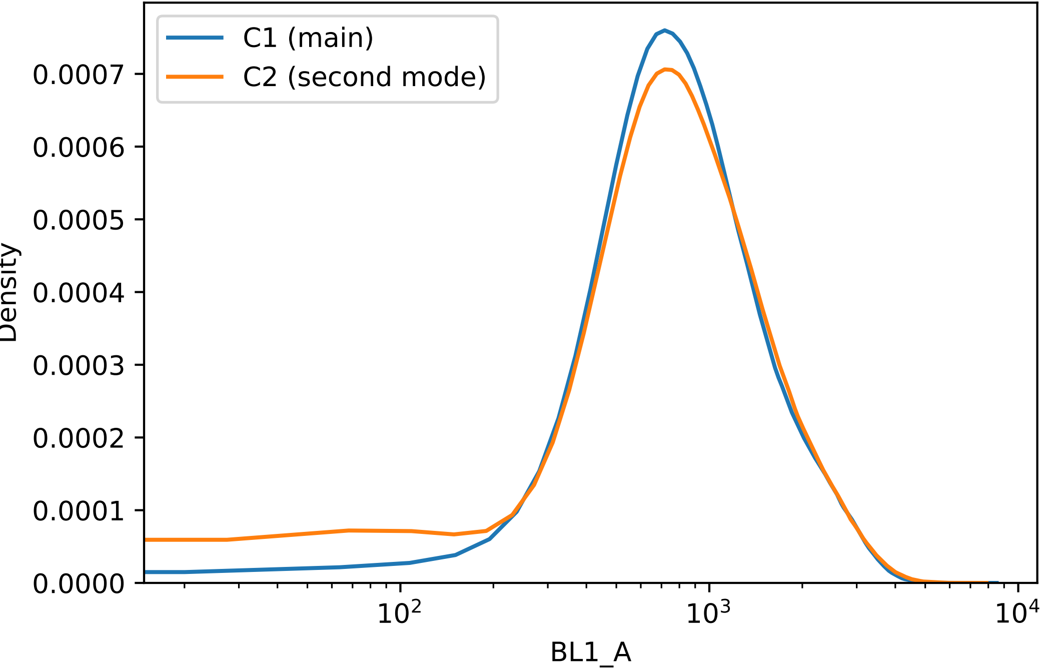

C1 (main)
C2 (second mode)
Density
0.0000
0.0001
0.0002
0.0003
0.0004
0.0005
0.0006
0.0007
10^2
10^3
10^4
BL1_A
