# Supplementary material for: Highly-automated, high-throughput replication of yeast-based logic circuit design assessments
Source: Synth Biol (Oxf). 2022 Oct 6;7(1):ysac018. doi: 10.1093/synbio/ysac018 (PMC9583850; doi:10.1093/synbio/ysac018)
Supplement: ysac018_Supp [file ysac018_supp.zip › suppl_data/SYNBIO-2021-042.tgz!/SYNBIO-2021-042/all_gate_replicates_normal_approx.pdf]

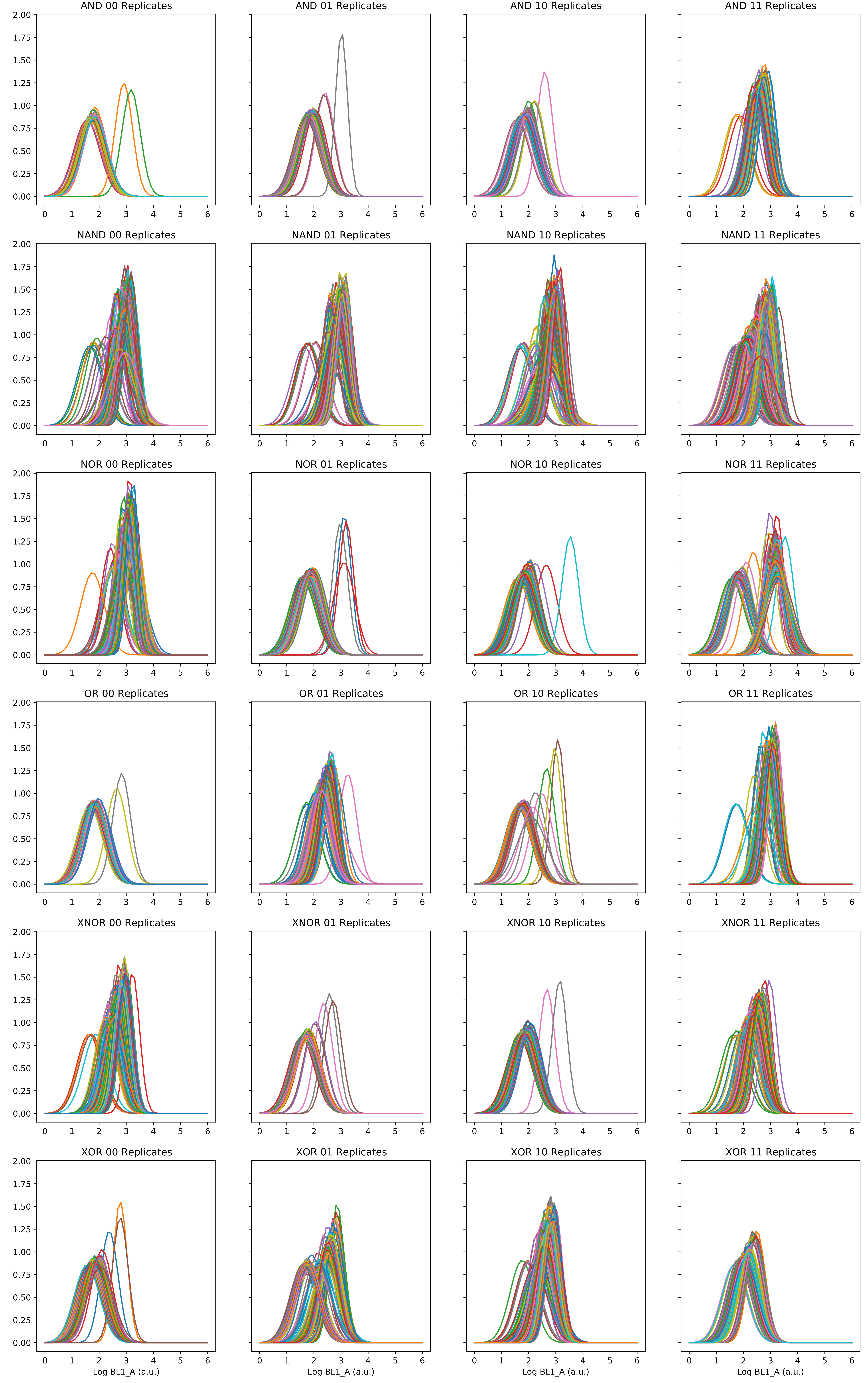
AND 00 Replicates
AND 01 Replicates
AND 10 Replicates
AND 11 Replicates
NAND 00 Replicates
NAND 01 Replicates
NAND 10 Replicates
NAND 11 Replicates
NOR 00 Replicates
NOR 01 Replicates
NOR 10 Replicates
NOR 11 Replicates
OR 00 Replicates
OR 01 Replicates
OR 10 Replicates
OR 11 Replicates
XNOR 00 Replicates
XNOR 01 Replicates
XNOR 10 Replicates
XNOR 11 Replicates
XOR 00 Replicates
XOR 01 Replicates
XOR 10 Replicates
XOR 11 Replicates
0.00 0.25 0.50 0.75 1.00 1.25 1.50 1.75 2.00
0 1 2 3 4 5 6
Log BL1_A (a.u.)
