# Supplementary material for: Highly-automated, high-throughput replication of yeast-based logic circuit design assessments
Source: Synth Biol (Oxf). 2022 Oct 6;7(1):ysac018. doi: 10.1093/synbio/ysac018 (PMC9583850; doi:10.1093/synbio/ysac018)
Supplement: ysac018_Supp [file ysac018_supp.zip › suppl_data/SYNBIO-2021-042.tgz!/SYNBIO-2021-042/growth_media/high_osmolarity.pdf]

Name	Measure	Units
L-Histidine	0.1	g/l
Adenine	0.08	g/l
ddH2O (sterile ultra-pure water)		
Dextrose (D-Glucose)	20	g/l
L-Tryptophan	0.1	g/l
D-Sorbitol	10	g/l
Thermo Scientific Remel Yeast Nitrogen Base w/o Amino Acids	6.7	g/l
DO Supplement -His/-Leu/-Trp/-Ura	1.4	g/l
Uracil	0.025	g/l
L-Leucine	0.1	g/l
