# Supplementary material for: Highly-automated, high-throughput replication of yeast-based logic circuit design assessments
Source: Synth Biol (Oxf). 2022 Oct 6;7(1):ysac018. doi: 10.1093/synbio/ysac018 (PMC9583850; doi:10.1093/synbio/ysac018)
Supplement: ysac018_Supp [file ysac018_supp.zip › suppl_data/SYNBIO-2021-042.tgz!/SYNBIO-2021-042/figures/preod_vs_postod_by_gate.pdf]

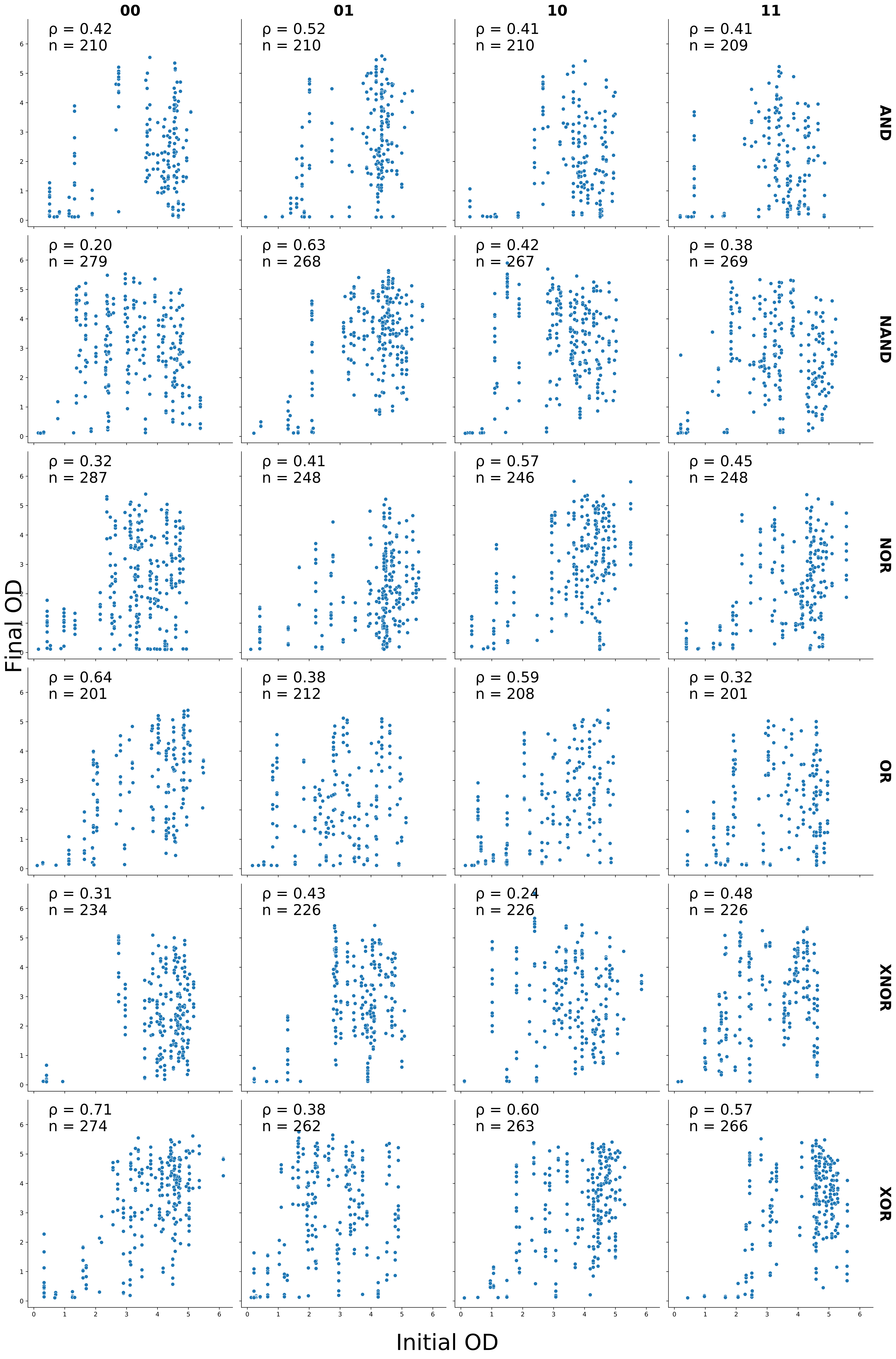
00
01
10
11
AND
NAND
NOR
OR
XNOR
XOR
ρ = 0.42
n = 210
ρ = 0.52
n = 210
ρ = 0.41
n = 210
ρ = 0.41
n = 209
ρ = 0.20
n = 279
ρ = 0.63
n = 268
ρ = 0.42
n = 267
ρ = 0.38
n = 269
ρ = 0.32
n = 287
ρ = 0.41
n = 248
ρ = 0.57
n = 246
ρ = 0.45
n = 248
ρ = 0.64
n = 201
ρ = 0.38
n = 212
ρ = 0.59
n = 208
ρ = 0.32
n = 201
ρ = 0.31
n = 234
ρ = 0.43
n = 226
ρ = 0.24
n = 226
ρ = 0.48
n = 226
ρ = 0.71
n = 274
ρ = 0.38
n = 262
ρ = 0.60
n = 263
ρ = 0.57
n = 266
Final OD
Initial OD
