# Supplementary material for: Highly-automated, high-throughput replication of yeast-based logic circuit design assessments
Source: Synth Biol (Oxf). 2022 Oct 6;7(1):ysac018. doi: 10.1093/synbio/ysac018 (PMC9583850; doi:10.1093/synbio/ysac018)
Supplement: ysac018_Supp [file ysac018_supp.zip › suppl_data/SYNBIO-2021-042.tgz!/SYNBIO-2021-042/figures/preod_vs_postod_by_inc_time.pdf]

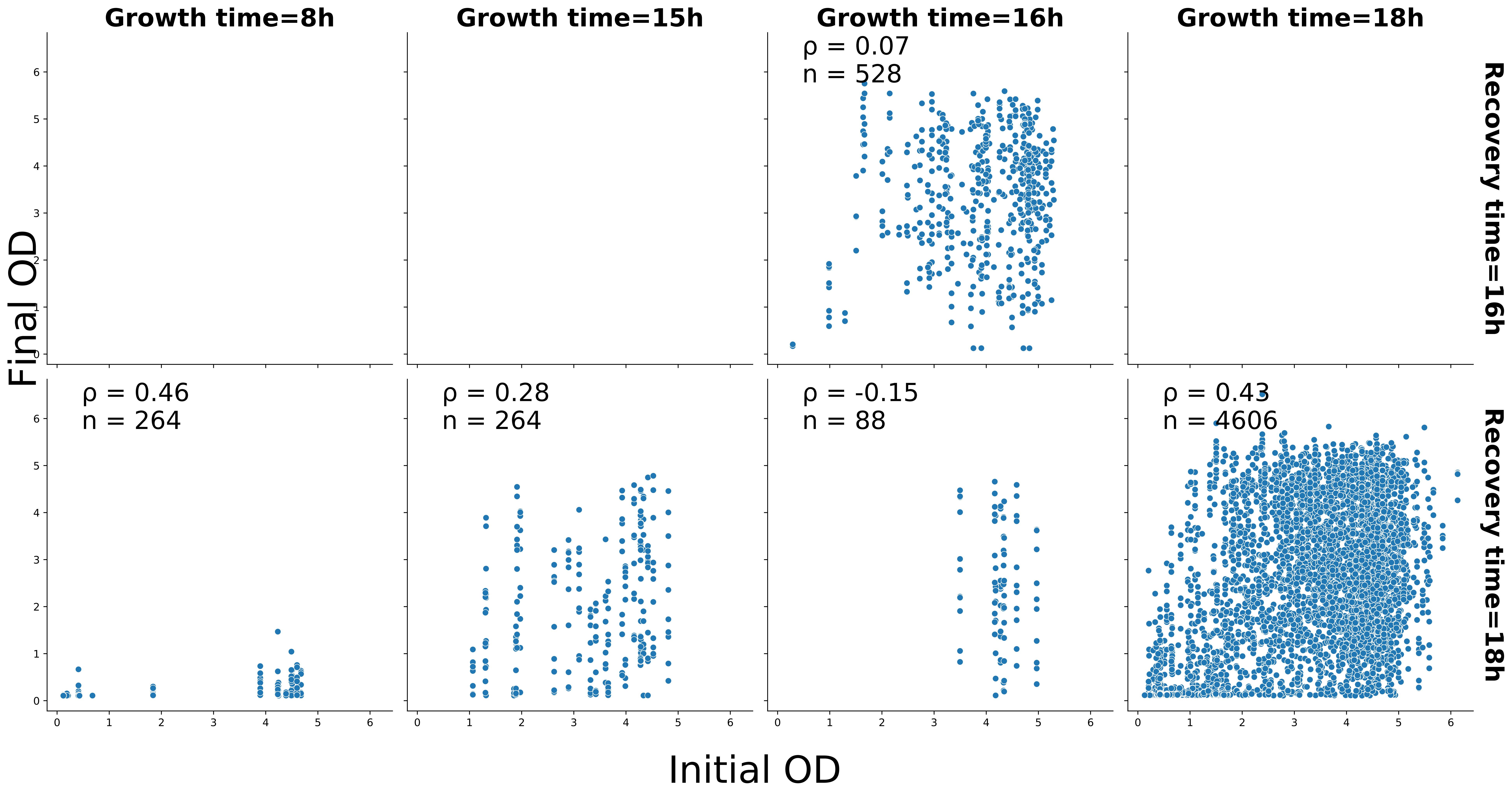
Growth time=8h
Growth time=15h
Growth time=16h
Growth time=18h
Recovery time=16h
Recovery time=18h
ρ = 0.07
n = 528
ρ = 0.46
n = 264
ρ = 0.28
n = 264
ρ = -0.15
n = 88
ρ = 0.43
n = 4606
Final OD
Initial OD
