# Supplementary material for: Highly-automated, high-throughput replication of yeast-based logic circuit design assessments
Source: Synth Biol (Oxf). 2022 Oct 6;7(1):ysac018. doi: 10.1093/synbio/ysac018 (PMC9583850; doi:10.1093/synbio/ysac018)
Supplement: ysac018_Supp [file ysac018_supp.zip › suppl_data/SYNBIO-2021-042.tgz!/SYNBIO-2021-042/figures/pre-post-corr-with-target-od.pdf]

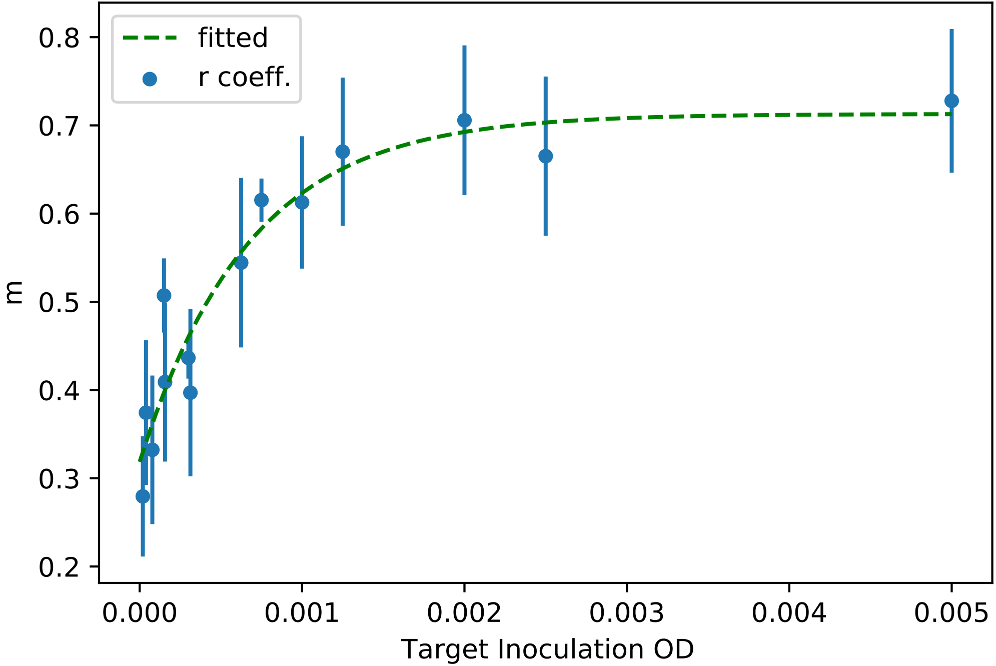

fitted
r coeff.
m
0.2
0.3
0.4
0.5
0.6
0.7
0.8
0.000
0.001
0.002
0.003
0.004
0.005
Target Inoculation OD
