# Supplementary material for: Highly-automated, high-throughput replication of yeast-based logic circuit design assessments
Source: Synth Biol (Oxf). 2022 Oct 6;7(1):ysac018. doi: 10.1093/synbio/ysac018 (PMC9583850; doi:10.1093/synbio/ysac018)
Supplement: ysac018_Supp [file ysac018_supp.zip › suppl_data/SYNBIO-2021-042.tgz!/SYNBIO-2021-042/figures/XOR_gate_designs_gRNA.pdf]

gRNA present								Design index
1	2	3	5	6	7	9	10	
1		1	1	1	1	1		1
1	1	1	1	1	1	1		2
1		1	1	1	1	1		3
1	1	1	1	1	1	1		4
1	1	1	1	1	1	1	1	5
1	1	1	1	1	1	1		6
1	1	1	1	1	1	1		7
1	1	1	1	1	1	1		8
1	1	1	1	1	1	1		9
1	1	1	1	1	1	1		10
1	1	1	1	1	1	1		11
1	1	1	1	1	1	1		12
1	1	1	1	1	1	1		13
1	1	1	1	1	1	1		14
1	1	1	1	1	1	1		15
